# Supporting meaningful contributions of people affected by Parkinson's throughout the research process: Key considerations for inclusive patient and public involvement across the globe

**DOI:** 10.1177/1877718X261502271

**Published:** 2026-10-27

**Authors:** Marie-Louise Zeissler, Kevin McFarthing, Michèle Bartlett, Natasha Fothergill-Misbah, Camille B Carroll, Ríona McArdle

**Affiliations:** 1 Faculty of Medical Sciences, Translational and Clinical Research Institute, 12186 Newcastle University, Newcastle upon Tyne, UK; 2 National Institute for Health and Care Research (NIHR) Biomedical Research Centre, Newcastle upon Tyne, UK 552379; 3 Expert through Experience, UK; 4 Population Health Sciences Institute, Faculty of Medical Sciences, 151476 Newcastle University, Newcastle upon Tyne, UK

**Keywords:** Parkinson's disease, patient and public involvement, lived experience, research participation, inclusivity, co-production, research life-cycle

## Abstract

Patient and public involvement (PPI), defined as research undertaken *with* people affected by a condition as partners rather than participants, is increasingly recognized as a marker of high-quality research and is now encouraged or required by many funders. Research agendas and practices need to reflect the realities of people living with Parkinson's Disease (PD) ensuring research addresses the priorities of those affected.

This narrative review examines the role of PPI across the research cycle, including priority setting, commissioning, study design, delivery, dissemination, implementation, and evaluation. We provide guidance and practical examples of how meaningful PPI can be achieved in PD research. The review explains how perspectives of lived experience can improve the relevance, feasibility, accessibility, and highlight its impact on PD research. We discuss practical considerations for embedding meaningful PPI, including project governance structures, dedicated staffing, remuneration, flexibility in conduct, and how to embed the perspectives of under-served PD communities in PPI. Finally, we consider challenges and opportunities for PPI in low- and middle-income countries, where underdiagnosis, stigma, health literacy barriers, and structural inequities require culturally responsive and community-led approaches.

Involving people affected by Parkinson's as equal partners in research has the potential to improve individual studies and to reshape the wider PD research agenda to be more inclusive, representative, and responsive to the priorities of those most affected.

To realise this potential, we must make a collective commitment to treat PPI as a requirement, provide funding and infrastructure support for implementation, and share experiences.

## Plain language summary title

How people affected by Parkinson’s can shape research

**Plain language summary:**

Patient and public involvement means doing research *with* people affected by a condition as partners, rather than doing research *to, about,* or *for* them (e.g. as participants). It is now widely seen as an important part of high-quality research and is encouraged or required by many organisations that fund research.

Many studies are not well aligned with the priorities or needs of those affected with Parkinson's, making findings less relevant and slowing research progress. This can only improve by involving people in every stage of the research cycle.

This review looks at how people with Parkinson's can contribute at every stage of the research process, including helping to decide which questions should be studied, reviewing funding applications, designing studies, helping run projects, sharing results, and evaluating where and how involvement has made a difference.

We describe evidence showing that lived experience can make research more relevant, practical, accessible, and useful. Involvement can also benefit contributors by increasing confidence, building new skills, creating social connections, and giving a sense of purpose.

For involvement to be meaningful, researchers need the right support systems in place: clear leadership, staff time, payment for expenses, flexible ways to involve people with lived experience, and efforts to include people from different backgrounds and communities.

We also discuss the unique challenges in low- and middle-income countries, where delayed diagnosis, stigma, low health literacy, and limited healthcare resources may make involvement more difficult.

Overall, treating people with lived experience as equal partners can improve individual studies and help create fairer, more inclusive research that focuses on what matters most to those living with the condition. However, we must make a collective commitment to support researchers and communities to achieve this.

## An introduction to involvement of people with Parkinson's in research

Parkinson's disease (PD) is among the fastest growing neurological disorders globally, with prevalence predicted to rise with population ageing.^1,2^ Despite advances in understanding, the aetiology and pathobiology of PD remain incompletely characterised, reflecting complex interactions between genetic and environmental factors. It is associated with a multitude of non-motor and motor symptoms that eventually lead to substantial disability, reducing quality of life, significantly impacting affected individuals, their families and healthcare systems.^3^

Clinical PD research presents distinct methodological and practical challenges. As a chronic, progressive neurodegenerative disorder that predominantly affects older adults, PD is frequently accompanied by multimorbidity, frailty and cognitive impairment. These factors are often insufficiently accommodated in trial designs. Additionally, under-served communities (defined as groups who face structural, social, or clinical barriers to accessing healthcare and research^4^) such as those from lower socio-economic or ethnic minority backgrounds, are generally underrepresented in research. Consequently, research is generally not representative of the real-world PD population.^5^ Studies may inadvertently exacerbate potential bias by design through restrictive eligibility criteria, prolonged follow-up periods and demanding visit schedules.^6,7^ Clinical trial attrition rates of 10–20% are common,^8^ with evidence indicating that withdrawal is more closely related to participant burden and protocol complexity than to treatment tolerability or safety.^7,9^ Beyond these operational challenges, research designed without the input of those it seeks to benefit, risks pursuing questions that are not relevant to everyday life with PD, limiting real-world impact and the uptake of findings into practice.

In 2022 Meinders and colleagues published a call to action appealing to the PD research community to include people affected by PD in study design and execution,^10^ aligned with increasing recognition of the importance of embedding patient and public involvement (PPI) into research practice more broadly. PPI should be standard practice in research and is a requirement for most funders of translational research in the UK including the NIHR, Parkinson's UK and Cure Parkinson's.^11–14^ PPI means doing research **with** people affected by a condition, not simply **to**, **about**, or **for** them.^15^ It encompasses a collaborative partnership, moving beyond interactions that are limited to data collection (i.e., participation). It is not only an ethical imperative that those affected by a condition should have a say in research^10^; real world, lived-experience perspectives can fundamentally improve the efficiency and value of research, focusing resources on questions that matter and improving transparency and impact of research.^16,17^

The benefit of PPI does not only extend to research practices but, if done well, can have a positive impact on PPI contributors and wider communities by increasing knowledge of research, promoting the development of new skills, building confidence, and giving a sense of empowerment.^18–20^

However, despite increasing consensus on the benefit of PPI, its widespread adoption globally remains challenging partly due to lack of infrastructure to support implementation.^21^ There are no reports that have attempted to synthesise existing evidence of PPI in PD research and thus, although emerging, evidence of PPI remains poorly documented in the literature. There is a lack of best practice examples, thorough evaluation of how PPI is conducted and its impact, or clear methodological guidance for implementation that is specifically tailored to PD.^10^ PwP have expressed a desire to be involved in shaping research^10,22,23^ and their involvement has tangible impacts on individual research projects and the wider research landscape.

In this review, we address where within the research cycle PPI adds value, providing PD-specific examples of impact. We offer practical guidance on embedding meaningful PPI in PD research, covering governance structures, PPI strategy and evaluation, staffing, budgeting, flexible approaches, and strategies to support the involvement of under-served groups. Finally, we consider the challenges faced globally for PPI in low- and middle-income countries (LMICs).

## Authors’ perspectives

The authors each bring distinct perspectives to this review. MB and KMF both have Parkinson's and have been involved in many local and international research projects including as participants, PPI contributors and co-researchers. MLZ and RMA are both early career researchers with experience in facilitating PPI across national and international research projects. RMA has delivered PPI across multiple neurodegenerative conditions including PD and across under-served communities. CBC is a neurologist and senior researcher who has led national working groups and initiatives to promote inclusive research and develop accessible research methods, particularly for clinical trials. NFM is an early career researcher with experience of carrying out Parkinson's research in LMICs, leading community engagement and involvement initiatives across seven countries in Africa and promoting awareness and advocacy of PD through co-designed documentaries, community theatre and other culturally-tailored methods.

## PPI in the research cycle

People with lived experience of PD can add value to all stages of the research cycle as outlined by the National Institute for Health and Care Research in the UK^24^ and the Parkinson's Foundation in the US,^25^ from shaping questions and securing approvals to conducting studies and disseminating findings and evaluating impact (Figure 1).^24^ Although PPI can be brought in at any stage of a project, we argue, the gold standard approach is to include PPI throughout. In this section we will consider each stage of the research cycle; PD specific examples of impact can be found in Table 1. It should be noted that for the purpose of this review these are focused on clinical research (i.e., studies that involve participants or sample collection) although there is also a growing recognition of the value of PPI in non-clinical research (e.g., laboratory research).^26^

**Figure 1.**
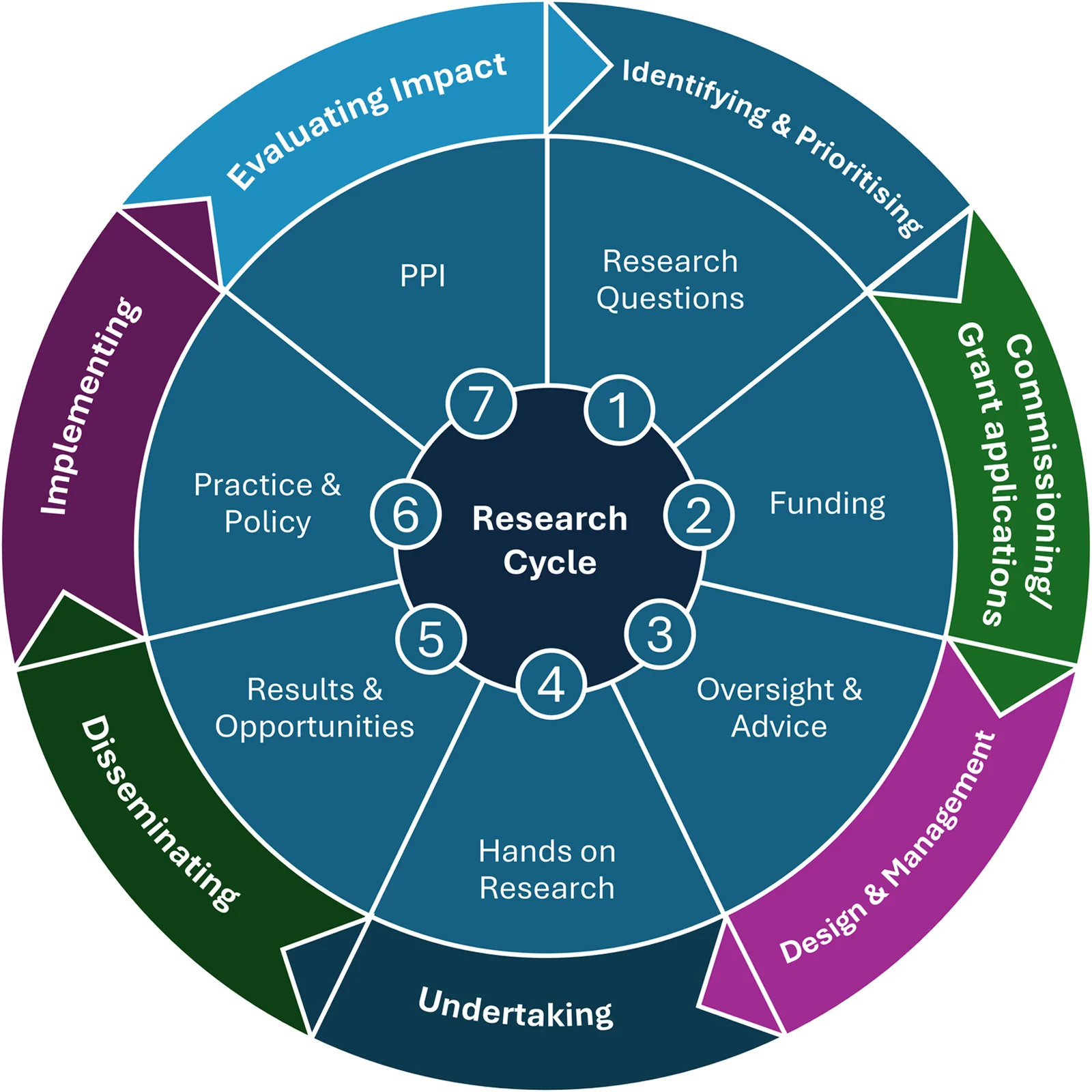
PPI in the research cycle. The outer ring depicts seven stages of the research cycle where PPI can get involved (adopted from the NIHR Briefing notes for Researchers and the NIHR Applied Research Collaboration West).^24,27^ The inner circle adds context to each stage.

**Table 1.** Examples of PPI impact on the research cycle in PD.

Research cycle stage	PD specific example	Impact
**Stage 1** Identifying and Prioritising	Strong advocacy by women in a virtual support group during the pandemic	A call to action and published review paper highlighting gaps in research and unmet needs in care of women with PD.^23,28^
	Community for Research Involvement and Support for PwP (CRISP), a patient-initiated group at King's College Hospital, was set up to discuss and review ongoing research, gather patient and public opinions on proposed projects and generate new research questions.^22^	The development of the King's Parkinson's Disease Pain Scale, arising from an unmet need identified through patient involvement.^29^
**Stage 2** Commissioning	Tom Isaacs, a PwP, founded the Cure Parkinson's charity and spearheaded the international Linked Clinical Trials (iLCT) initiative^30^	iLCT is still active today, has prioritised disease-modifying therapies for the past 14 years and played a pivotal role in expanding the drug development pipeline, catalysing clinical trials including platform trials.^31–33^
**Stage 3** Design and Management	The PPI working group of a UK research delivery network (DeNDRoN) advised on MUSTARDD-PD, a multicentre UK study of the acetylcholinesterase inhibitor donepezil in early PD dementia encountered difficulties with broaching the subject of dementia with PwP appropriately and sensitively.^34^	Appropriate language to introduce the subject of dementia was co-developed. The word dementia was used less to mitigate anxieties linked to negative connotations with this word. It was replaced by terminology which pertained to the range of potential symptoms and signs, was more sympathetically worded, and encompassed differing aspects of the problem.^34^
	The Edmond J Safra Accelerating Clinical Trials in Parkinson's initiative has involved PwP in all aspects of the development of the first multi-arm multi-stage platform trial, with PPI embedded at project and steering group level.^35^	Informed all aspects of study design. It led to an emphasis on the development of an inclusive study with minimal exclusion criteria.^36^ A patient-centred primary outcome was selected.^37^ PPI representatives contributed throughout the treatment selection process, informing practicalities, side-effect acceptability, and tolerability criteria, with lay dossiers produced for their review.^38^
	The Mobilise-D programme exemplifies PPI co-production across the project's lifecycle. A Patient and Public Advisory Group was integrated into the project's governance structure, co-producing a PPI strategy and action plan.^39^	Input directly influenced how digital mobility data were defined and presented. It showed that real-world walking was a meaningful outcome to measure and led researchers to adapt outputs (e.g., improving accessibility of data displays and ensuring results reflected lived experience).^39^
**Stage 4 **Undertaking	Annual clinical pipeline review of drug therapies led by a PwP.^31^	Highlights increasing clinical research activity each year.
	A mixed methods study of the benefits and burdens of self-tracking for PD, led and conducted by a PwP.^40^	Proposal of a model for self-tracking for PwP facilitating a deeper understanding of their own disease and contributing to more effective decision making.
	Participatory co-research in a collaborative qualitative Parkinson's dance project^41^	Highlighted the benefits of reflexive analyses of power-dynamics in participatory research to break down perceived power-imbalances.
**Stage 5** Disseminating	Powerful narratives, led or co-led by people with lived experience of PD.	Have helped to raise awareness of research gaps and lent greater focus to health care disparities for Women in PD^28^ Young onset PD (YOPD)^42^ Non-motor symptoms^43^ Importance of patient centred approaches in gene and cell therapy trials^44^
	Co-produced videos to explain research procedures such as MRIs^45^ or lumbar punctures^46^	Improved understanding and increased willingness to participate in research involving these procedures.^47^
**Stage 6** Implementing	A PPI programme running along-side the PPMI initiative^47^	The myPPMI online portal was created to simplify study participation, introducing participant-requested return of personal research results.^47^
	Following co-design of the Home Based Care (HBC) pathway, people with Parkinson's and care partners continued as members of the HBC service implementation and leadership group, working alongside clinicians and managers to oversee rollout and ongoing refinement of the pathway.^48,49^	Ensured that implementation of the co-created care pathway remained grounded in lived experience, with PwP helping to decide how the service was delivered, adapted and scaled in routine practice, rather than the model reverting to a purely clinician-led design.^48^
**Stage 7** Evaluating Impact	Examples of evaluation of PPI in PD projects include the PRIME UK project,^50^ the EJS ACT-PD initiative^35^ and the Mobilise-D consortium^39^	Improved understanding on the effective conduct and impact of PPI, such as how to build effective government structures^35,39^

### Stage 1 identifying and prioritising

PwP, researchers and clinicians may have distinct but overlapping priorities and views; partnerships enable these to be aligned into shared research agendas grounded in lived experience. This is an important but often not sufficiently practiced aspect of PPI. Involvement of people affected by Parkinson's right at the beginning of projects ensures research is relevant by helping identify research topics.

### Stage 2 commissioning

Many funding organisations now involve patients and the public in commissioning research to ensure funding decisions reflect patient priorities and perspectives. This involvement can include serving on funding panels, contributing to the commissioning of research and monitoring of funded studies. In research projects, PPI contributors may review proposals before submission, or be co-applicants on a research proposal.^51^

### Stage 3 design and management

PPI in research design ensures studies are relevant, ethical, meaningful and feasible by setting realistic study participant expectations and is a requirement for most funders in the UK. Lived experience contributors play a key role in shaping the structure and protocol of the study, developing participant-facing information, and helping to communicate risks, uncertainties, and expectations in a clear and meaningful (e.g., to support informed consent). Whilst invaluable at the study design stage,^50^ PPI in study management ensures effective and responsive problem solving that is feasible and acceptable to study participants. In PD research, participatory approaches increasingly position PwP as active partners in the design and conduct of studies.^35,39^

### Stage 4 undertaking

Members of the public can also act as co-researchers, taking on a range of roles in conducting research, including collecting and reviewing evidence, and undertaking literature-based work. Public contributors can add an additional lens to qualitative studies through conducting interviews and focus groups, and contributing to the analysis and interpretation of findings.^52,53^ Involving lived experience co-researchers in qualitative research projects is particularly valuable as it generates invaluable insights, highlighting assumptions and reducing interpretive bias.^53^ In quantitative research, PwP can contribute to determining which variables and outcomes are most meaningful from a lived experience perspective, support interpretation of findings through the lens of lived experience, and assist with data collection where appropriately trained. Researchers can also employ a participatory research or participatory action research approach whereby PPI contributors are both participants and co-researcher.^54,55^ However, careful methodological consideration is needed to address power-dynamics and enable equal weight and contribution across all co-researchers.^41^ Reflexive analyses that deliberately acknowledge, review and address perceived power-imbalances can be an effective strategy to break down hierarchies.^41^

### Stage 5 disseminating

PPI in dissemination helps ensure research findings are communicated clearly, reach wider audiences, and are more likely to influence practice, particularly when contributors have been engaged throughout the project and feel a sense of ownership. Practical approaches include providing regular updates, co-developing dissemination plans, involving public contributors as presenters or co-authors, using their networks to extend reach, and co-producing accessible summaries, videos, and other public-facing outputs. PPI in community based dissemination activities can also be an effective tool for enhancing diversity and promoting health equity in PD research by demonstrating impacts of research in communities strengthening reciprocal relationships.^56,57^

### Stage 6 implementing

PPI can strengthen the translation of research into practice by helping to drive meaningful change in areas that matter most to patients and communities. Public contributors are often highly motivated to see action from research findings and can play a direct role in driving implementation: using their networks and relationships with patient organisations, clinical teams, and policymakers to support uptake; co-designing implementation strategies that reflect real-world barriers; and serving as advocates and champions within their own communities. Their continued involvement at this stage can help ensure that the changes arising from research genuinely improve the lives of PwP.

### Stage 7 evaluating impact

Sharing approaches to PPI, their successes, challenges and impact is essential for building the evidence base for successful PPI in Parkinson's research.^10^ A range of tools exist to support the evaluation of PPI, as discussed in the PPIE strategy and evaluation section below. Researchers should report how community input influenced decisions, learn from failures, and treat accountability as essential to meaningful involvement. Transparent reporting using the GRIPP-2^58^ reporting guidelines is recommended.

## Key considerations for embedding PPI in PD research

There are multiple tools and guidance frameworks that can help inform researchers on how to conduct PPI effectively with the aim to improve the quality and consistency of public involvement in research.^59^ These include the UK standards for public involvement which consist of six core elements to consider: governance, working together, inclusive opportunities, communication, support & learning, and impact.^60^ Embedding PPI meaningfully requires careful consideration and forward planning of governance frameworks, PPIE strategy and evaluation, budget and resources, dedicated staffing, flexibility in project activities, and provisions to ensure inclusivity across the process. It is vital to acknowledge that ‘one size does not fit all’ when it comes to PPI, and researchers must be responsive to changing needs and priorities of PPI contributors. This section provides an overview of key considerations with suggested actions to support meaningful PPI in Parkinson's research.

### Governance framework

A core principle of the UK Standards for Public Involvement is that PPI should be embedded in research management, leadership, and decision-making. A common mechanism is a patient and public advisory group.^61,62^ These groups enable contributors to share views, provide feedback, and act as catalysts for change within the research process.^39^

Their positioning within project governance depends on project scale. In smaller studies, the entire research team may work directly with PPI contributors. In larger projects, such as clinical trials or multi-workstream programmes, contributors may sit on trial management groups, steering committees, or in work-package meetings.^35,39^ The position of the advisory group within the overall governance structure has a significant bearing on whether contributors feel able to make a meaningful contribution; positioning PwP at the level of key decision-making bodies, rather than as an add-on layer of consultation, is central to genuine partnership. However, representation alone is insufficient. Contributors must be supported to participate meaningfully.^63^ This may include providing annotated papers in advance, using plain language, avoiding jargon, and allowing time for reflection and response. Support needs should be reviewed throughout the project.^10^

It is crucial that both the patient and public advisory group and the research teams understand each other's roles and responsibilities as well as a clear definition of the project remit, including what is feasible and what lies beyond scope.^59^ Developing formal ‘Terms of Reference’ that are agreed early and refined with group input help establish these principles. These should cover the group's purpose, membership, meeting frequency, communication methods, remuneration, confidentiality, and how decision-making power will be shared. A code of conduct can promote respectful working, for example an agreement to use person-first language and avoid disempowering or disability-focused terms (e.g., ‘people with Parkinson's’ rather than ‘Parkinson's patient’).

A project induction that clearly sets out the premise for PPI and outlines what will be involved in the project is essential for building a joint understanding of the project from the outset. Providing ongoing training, support, and clear information throughout a project is essential to enable PPI contributors to participate confidently and make meaningful contributions.^59^

### Developing a PPI strategy and evaluating impact

Meaningful PPI requires deliberate planning, clear documentation, and systematic evaluation. Developing a PPI strategy at the outset of a project, ideally co-produced with contributors, provides a roadmap for how involvement will be embedded across the research cycle. This creates the foundation for accountability and learning.^10,64^

A well-developed PPI strategy should set out the aims and scope of involvement, the stages at which PPI will occur, the roles and responsibilities of contributors and the research team, governance arrangements, and the resources allocated. It should also articulate from the outset how involvement will be evaluated, covering contributor experience, processes, and impact. Importantly, it should describe how findings will be fed back to contributors and the wider research community. The strategy should be a living document, reviewed and updated with contributors throughout the project.^35,39^

Evaluation of PPI operates at three distinct but interconnected levels:

**Level 1** is contributor experience: whether those involved found the experience meaningful, well-supported, and worth their time. This is both an ethical obligation and a practical one. Poor experiences undermine future recruitment and damage trust.^65^ Structured feedback through post-meeting questionnaires, one-to-one conversations, or validated tools such as the Patient Engagement in Research Scale (PIERS)^66^ can capture this systematically. Feedback should be acted upon visibly, with contributors informed of changes made in response.
**Level 2** is process evaluation: whether PPI was conducted in a way that was accessible, inclusive, and fit for purpose. This includes examining whether meetings were accessible to contributors with PD-related symptoms, whether materials were in plain language, whether diverse groups were reached, and whether contributors genuinely influenced decisions. Reflective tools such as Public Involvement Impact Logs^67^ and the Cube Evaluation Framework^68^ can support this ongoing self-assessment.
**Level 3** is impact evaluation: documenting the tangible difference PPI made to the research, encompassing changes to study design, protocol, outcome measures, recruitment materials, dissemination, and research priorities. The Public Involvement Impact Assessment Framework^69,70^ provides a structured approach to capturing this.

In PD research, examples of well-evaluated PPI, including from the PRIME-UK project,^50^ the EJS ACT-PD initiative,^35^ and the Mobilise-D consortium,^39^ demonstrate that impact evaluation is feasible even within large, complex programmes.

Findings from PPI evaluation should be reported and shared. The GRIPP-2 reporting guidelines^58^ provide a standardised framework for transparent reporting of PPI in publications and their use should be considered routine practice. The current evidence base for PPI in PD research remains limited,^10^ and every well-documented example contributes to building it.

### Budgeting PPI activities

Meaningful PPI is not cost-free and requires recognition of the time and financial demands placed on both researchers and contributors.^11,71^ In the UK, NIHR guidance promotes a consistent approach to remuneration and recognition.^72^ It outlines guidelines for remuneration including payment for time, training, and dissemination, alongside reimbursement for travel, childcare or carer costs, and subsistence, as well as remote working costs (e.g., contribution for internet use).^71^ Additional costs may include researchers’ travel to communities, relationship-building, training, venue hire, interpretation, catering, and conference attendance for contributors.^71^

A modest contingency fund within a project budget can help ensure that practical changes recommended by contributors can be incorporated without requiring a budget amendment. NIHR payment guidance^72^ provides a useful framework for costing PPI activities and supports realistic project budgeting. It is crucial that project payment policies are transparent and ideally co-developed with contributors to ensure arrangements are practical and do not adversely affect welfare benefits. The latter point is critical for inclusive involvement presenting a specific barrier for under-served groups. Some contributors may not be eligible for standard financial remuneration, for example those without the right to work, and so alternative forms of recognition, such as reimbursement for shared food, conference invitations, or access to training, should be considered.^73^ University reimbursement processes are often slow and bureaucratic.^74^ Researchers should assist contributors in navigating these where possible. Awareness of these issues should be actively raised with institutional and funding bodies to promote standardised, equitable solutions.

While setting standards for involvement and engagement throughout the research cycle is important, the expectation that PPI is fully funded from the outset risks disadvantaging researchers who lack access to dedicated funding streams. This barrier is felt most acutely by early career researchers, who are less likely to have established funding networks or the track record needed to secure competitive grants that accommodate PPI costs.^75^ Encouragingly, some funders already offer dedicated funding streams to support PPI.^76^ Ad hoc PPI input, while not ideal, can still add value to research; however, it should not become the default outcome of inadequate resourcing. Funders should therefore be encouraged to develop accessible, dedicated funding streams that support meaningful PPI across all career stages.

### Dedicated staff resources

Building and sustaining a PPI group requires substantial time and staffing, often underestimated in research proposals.^77^ The appointment of a dedicated PPI Lead is recommended, with time allocated according to project size, to support contributors and embed their perspectives across the study.^11^ The PPI Lead often takes on ‘invisible’ but crucial work for the conduct of meaningful PPI. Key responsibilities include developing partnerships, recruiting and supporting contributors, maintaining communication and records, managing budgets, ensuring governance and data protection compliance, translating materials into plain language, liaising between PPI contributors and researchers, and feeding back outcomes.^11,77^ PPI Leads need appropriate capacity, interpersonal skills, training, and administrative support.^11^

### Flexible approaches to support the changing needs of PwP

Embedding PPI across the research cycle requires sustained commitment from PwP, care partners, and health or social care contributors. Long-term projects benefit from some continuity of membership to maintain discussion, priorities, and knowledge mobilisation.^39^ However, Parkinson's is progressive^4^ and significantly affects health and wellbeing,^78^ with significant impacts on PwPs and their care partners and families. Thus contributors’ capacity to engage may fluctuate over time. Some may pause or end involvement, and this should be anticipated and supported.^39^ Peer mentoring or shadowing arrangements, whereby established contributors support incoming members, can help preserve institutional knowledge and maintain continuity of the group's priorities and expertise as membership evolves.

Researchers or PPI Leads should work with contributors to identify how they can remain involved in ways that suit their needs and preferences.^10^ PwP may experience changes in communication, including slower processing, speech difficulties, or word-finding problems, which can sometimes make it more challenging to follow or contribute to conversations, particularly in group settings.^79^

Offering multiple routes to involvement can improve accessibility, such as smaller meetings, one-to-one conversations, written feedback, remote participation, movement breaks, and flexible scheduling around symptom fluctuations.^10^ Creative methods, including arts-based or visual approaches, may also help capture non-verbal perspectives and keep contributors central to the process.^80^

Cognitive decline is common in Parkinson's with estimates of 22–66% of PwP having mild cognitive impairment^81^ and 26% with Parkinson's disease dementia.^82^ Cognitive impairment comes with unique barriers to PPI for PwP that require both mitigation and thoughtful adaptation. Attitudinal barriers, such as assumptions that people with cognitive impairment cannot contribute meaningfully, or gatekeeping by carers and clinicians, must be actively challenged and mitigated. Practical barriers, including difficulties communicating complex information and inflexible involvement methods, require careful adaptation of approaches to ensure meaningful participation is possible.^83^ Evidence from dementia PPI suggests effective approaches include person-centred planning, allowing contributors to shape methods and level of involvement, using small or one-to-one meetings in accessible settings, building rapport, and training researchers in cognitive impairment.^63,83^ Addressing attitudinal barriers requires deliberate effort, including challenging assumptions about capacity and actively working to reduce gatekeeping by carers and clinicians. Above all, PPI should remain flexible, strengths-based, and responsive to changing needs.^80^

### Inclusivity

In PD research, under-served communities include people from ethnic minority or low-income backgrounds, those living in rural or remote areas, women, people with young-onset PD, those identifying as LGBTQIA+, those living in nursing homes and people living with cognitive impairment or dementia.^5,84^ Within the PD context, additional factors such as atypical symptom presentation, stigma, and inappropriate support structures can compound these barriers, contributing to delays in diagnosis and underrepresentation in research.^28,42,85–87^ Critically, it is not for the researcher alone to determine who is under-served; partnership with diverse communities should inform the identification of representation gaps and guide efforts to promote inclusion.^88^

Involving under-served groups in PPI goes far beyond improving recruitment. Their perspectives are essential to ensuring that the research agenda, the questions asked, and the methods used are genuinely relevant to the full spectrum of people living with PD.^73,89^ Without these voices, research risks perpetuating the very inequalities it aims to address.^84^

Reaching under-served communities requires proactive, sustained outreach. Researchers should build links with community networks including religious organisations, community care initiatives, social clubs, and outreach programmes.^74^ These relationships must be reciprocal: research teams should invest in the communities they seek to involve, offering support in return (e.g., running health education events, assisting with grant writing, or facilitating community-identified research activities). This builds rapport with trusted community members, often described as gatekeepers and is central to sustaining these partnerships.^74,90^

Recruitment materials and PPI structures must also be examined critically. Advertising should explicitly signal the desire to involve people from specific under-served groups and use imagery that reflects diverse ages, ethnicities, and abilities. Formal PPI structures such as advisory groups can inadvertently exclude under-served groups by assuming high health literacy, by scheduling meetings only during working hours, or by requiring in-person attendance.^88^ Offering flexible meeting times, smaller group or one-to-one formats, remote and mobile participation options, and accessible plain-language materials can mitigate these barriers, though care should be taken to avoid digital exclusion.^91^

It is important to acknowledge, however, that the strategies outlined above can only reach those who already have a diagnosis. Many under-served groups face barriers to help-seeking and acquiring a diagnosis,^28^ and thus remain invisible to research. However, these groups’ perspectives are valuable assets to informing Parkinson's research, and we must consider how to build trust, awareness and advocacy within these communities to allow them to share experiences and set priorities. Moving away from traditional advisory models of PPI towards community engagement and involvement methods^92^ can build these trusted relationships and bring the voice of these under-served groups into the research process.

### Challenges to effective PPI and mitigation strategies

Despite the clear value of PPI, embedding it meaningfully in PD research is not without difficulty. Challenges arise at the level of individual contributors, research teams, and institutions, and are often compounded by the specific features of PD as a progressive, heterogeneous condition. Table 2 summarises key challenges and practical strategies to address them.

**Table 2.** Common challenges to effective PPI in PD research and suggested mitigation strategies.

Challenge	Mitigation strategies
**Project governance**	
Insufficient knowledge about the project and proposed PPI roles	Conduct project induction and training forums to increase mutual understanding;^59^ define the scope and objectives for activities; develop activity plans with clear aims, methods, and responsibilities; include PPI roles in Terms of Reference for working groups and committees.^35,39^
Tokenistic rather than genuine involvement	Define decision-making power in Terms of Reference; ensure contributors attend key decision points; ensure PPI is embedded within meeting agendas; feedback to contributors how their input influenced the research.^10,59^
Power imbalances between researchers and contributors	Use reflexive analyses to acknowledge and address hierarchies; position contributors within formal governance structures; consider participatory approaches.^41^
Lack of researcher training and awareness	Embed PPI training in researcher development; draw on lived experience contributors as educators; foster a culture that values PPI as integral not additional.^10,18^
**Understanding impact**	
Insufficient evaluation and reporting	Develop a PPI strategy with evaluation built in; use validated tools at contributor, process, and impact levels; report using GRIPP-2.^58,64,66,69,70^
Input not visibly acted upon	Maintain clear records of PPI influence on decisions; provide regular feedback to contributors; report impact using GRIPP-2.^58^
**Resourcing**	
Inadequate budgeting and resource	Budget for PPI from the outset including staff time, remuneration, travel, and a contingency fund; follow NIHR payment guidance; involve contributors in developing payment policies.^11,71,72^
Underestimated staff time	Appoint a dedicated PPI Lead with protected time proportional to project size; recognise the breadth of invisible PPI work in project planning.^11,77^
Institutional barriers to remuneration	Streamline reimbursement processes; assist contributors with expense forms; consider alternative recognition for those ineligible for financial payment.^73,74^
**Supporting needs to PPI contributors**	
Cognitive impairment and communication difficulties	Use small or one-to-one meetings; provide plain-language materials in advance; allow extra processing time; consider arts-based methods; train researchers in communicating with people with cognitive impairment. ^63,79,80,83^
Progressive disease affecting contributor capacity	Plan for membership turnover; offer flexible stepped involvement; implement peer mentoring or shadowing schemes to support knowledge transfer when new members join.^39^
Contributor fatigue or feeling out of their depth	Provide induction and ongoing training; use plain language; offer briefing documents before meetings; check in regularly on wellbeing and experience.^10,63^
**Inclusivity**	
Under-representation of diverse and under-served groups	Proactive community outreach; community engagement and involvement methodologies;^92^ reciprocal partnerships with trusted community organisations; flexible meeting formats and times; targeted recruitment materials.^73,88^

## Global inequalities, cultural context, and meaningful involvement in PD research

Despite increasing evidence of the growing burden of Parkinson's worldwide,^93^ and despite almost half the population of PwP living in LMICs, research has remained concentrated within high income countries (HICs) and dominated by Western narratives.^94^ This persistent under-representation of PwP from LMICs not only reflects an exclusion from research opportunities which limits knowledge of disease aetiology and restricts the applicability of advances to broader populations, but also reflects the broader inaccessibility of diagnosis and treatment for PD globally. PPI in a global context must acknowledge the complex dynamics of power and privilege that shape research in LMICs.^95^ As such, PPI as conceptualised in HICs does not directly translate to LMICs.

In many LMIC settings, where underdiagnosis is widespread, stigma profound, and health literacy limited, Community Engagement and Involvement (CEI), a broader concept than PPI that foregrounds awareness-raising, advocacy, and trust-building,^96^ may be a more appropriate and achievable starting point than conventional PPI frameworks.^92^ CEI does not position PwP as experts by experience in the way HIC PPI models do; rather, it creates the conditions in which people can first access diagnosis and community, and from there potentially move towards more formal research involvement. This distinction is important and should be reflected in how researchers approach involvement in LMIC settings.^97,98^

Despite its importance, the evidence base for PPI in PD research, and indeed health research in LMICs more broadly, remains sparse.^97^ A systematic review found that PPI strategies and impacts are rarely documented in health research in LMICs.^99^ Without documentation or reporting, PPI and engagement risks being tokenistic, particularly considering the structural constraints distinctive to many LMIC settings. For example, paternalistic relationships between patients and healthcare professionals,^100^ health literacy challenges and entrenched multi-level stigma around neurological conditions like PD.^101^ Navigating these conditions requires researchers to go beyond adapting HIC derived PPI frameworks and to engage in genuinely reflexive, culturally and context-sensitive practices.

### The challenge of power and privilege

PD research, like global health research more broadly, has historically been dominated by institutions in the Global North. Research agendas and methodologies have been set by funders and academic institutions in HICs, with LMICs often positioned as sites of data collection rather than co-generators of knowledge. Decolonising global health research requires institutions in HICs to acknowledge and relinquish power and authority,^102^ though such legacies are difficult to dismantle. Applied to PD PPI, this means critically examining research priorities, agendas and methodologies that are often adopted from HICs. Further, in the context of PD research, where diagnosis and treatment access are already precarious, and where healthcare professionals are potentially gatekeepers of knowledge and medication, the hierarchy between PwP and medical professionals is pronounced. PPI models that place the voices of people with lived experience of PD at the centre of the research process may therefore represent ‘uncharted waters’^100^ for many healthcare professionals in LMIC settings. PPI may also be seen as ‘extractive’, rather than truly involving communities.^103^ This risk is heightened when PPI is not supported by appropriate investment in time, trust, resources, and decision-making power. This re-iterates the need for broader CEI approaches.

### The challenge of health literacy challenges and marginalisation

Communities are not homogeneous, and within LMIC populations, the most marginalised individuals are often least likely to obtain a diagnosis of PD, excluding them from any further opportunities for engagement. Those who have never received a PD diagnosis, likely the majority of PwP in many LMIC settings, are effectively invisible to PPI.

Health literacy shapes who can participate in PPI and how activities are designed. In many parts of the world, PD does not have a recognised name and is often described simply as ‘the shaking illness’, as evidenced from East Africa, for example.^104^ The condition is frequently associated with supernatural explanations which result in delayed diagnosis and stigma.^105^ Such perceptions have direct implications for PPI in that stigma affects individuals’ willingness to be identified as having PD and to engage in research.

Identifying and reaching PwP in this context requires creative, proactive and sustained engagement with local communities, whether through more structured methods (e.g., community-based door-to-door prevalence studies^106^) or through community awareness-raising initiatives (e.g., TV and radio shows or engagement with religious leaders).

In addition, a basic level of understanding about PD and its symptoms cannot be assumed. Starting PPI processes with culturally sensitive orientation to PD and without dismissing local explanatory models is therefore a prerequisite. Accessible and multilingual materials are crucial when considering literacy levels, while digital literacy and the availability of internet connectivity are additional considerations.

### Adaptation of research tools and processes

PwP and their care partners or families should be involved in shaping culturally appropriate consent processes, outcome measures, and data collection tools. Standard research instruments, including many PD rating scales and quality-of-life measures, have been developed and validated primarily in HIC populations and often do not translate culturally or linguistically, nor do they capture the dimensions of experience most relevant to PwP in LMIC settings. Community input through CEI is therefore essential in identifying these gaps and supporting the co-development of tools that are contextually meaningful and applicable.

Community input is also critical in designing consent processes that acknowledge language, literacy and cultural norms. Across Africa, consent can be a communal process,^107^ where permissions may be needed from a gatekeeper or wider family, this can prove difficult if PwP are wary of sharing a diagnosis for fear of stigma. The process of seeking consent must be handled with cultural sensitivity, explaining clearly what research involves, why it is being conducted, and what community members can expect to receive in return. The question of remuneration also requires local ethical deliberation, balancing the obligations of fairness against the risk of undue inducement in contexts of economic precarity.

The TraPCAf programme exemplifies how CEI can be embedded in Parkinson's research across sub-Saharan Africa.^108,109^ TraPCAf has prioritised awareness-raising, trust-building, and partnership with local communities as an underlying tenet for its research programme. The programme works with PwPs, care partners, clinicians, and community stakeholders to shape research priorities and improve care pathways. Drawing on the TraPCAf experience and CEI principles, we make recommendations for integrating considerations of power imbalances, inequities in access to care, and health literacy challenges into PPI in LMIC contexts (Table 3).

**Table 3.** Recommendations for PPI in the LMIC context.

Action	Recommendations
Act at the earliest stage	Identify locally relevant priorities such as access to levodopa, traditional medicine, care partner burden, and diagnostic delay.
	Build trust through sustained relationships, particularly where extractive research practices have caused harm.
Redesign recruitment, participation and methods	Develop strategies to identify and include PwP who remain undiagnosed or disconnected from care.
	Create safe, stigma-sensitive opportunities for involvement that do not require public disclosure of diagnosis.
	Choose or create culturally appropriate consent processes, outcome measures, and data collection tools.
	Determine ethical and fair remuneration.
Share power in analysis and interpretation	Involve PwP and care partners in reviewing findings so results reflect local realities rather than assumptions imported from high-income settings.
	Provide accessible materials and clearly document how community input shaped conclusions.
Disseminate for impact, not just publication	Return findings to participating communities, frontline healthcare workers, and policymakers through accessible, multilingual outputs.
	Support PwP as advocates who can raise awareness, reduce stigma, and influence policy.
Invest beyond individual projects	Short-term PPI risks exploitation. Funders must support lasting infrastructure, including support groups, patient organisations, local leadership, and researcher training.

## Conclusions

The meaningful involvement of people with lived experience of PD across the research cycle is no longer optional but essential to delivering high-quality, equitable and impactful Parkinson's disease research. PPI shifts research from being conducted about PwP to being undertaken with them, ensuring that priorities, methods and outcomes reflect real-world needs and lived experience. Across all stages of research, lived experience brings perspectives that can improve relevance, reduce participant burden, strengthen trust and enhance translation into practice.

However, meaningful PPI requires more than goodwill. It depends on investment in infrastructure to support dedicated staff time, fair remuneration, inclusive governance, accessible communication and robust evaluation. It also requires reflexivity from researchers, with willingness to share power, challenge assumptions and adapt methods to contributors’ changing needs. Importantly, approaches developed in high-income countries cannot simply be transferred globally; culturally responsive, community-led models are needed, particularly in LMIC settings. The distinctive features of the LMIC PD context of including widespread underdiagnosis, multi-level stigma, power imbalances, low health literacy, and scarce specialist infrastructure create an urgent need for meaningful community engagement initiatives that drive locally relevant research.

Ultimately, embedding people with lived experience as equal partners offers an opportunity not only to improve individual studies, but to reshape the Parkinson's research landscape into one that is more democratic, representative and responsive to those most affected.

Realising this potential requires collective commitment. We call on funders to make PPI a funded requirement across all stages of the research cycle and to develop accessible mechanisms for early career researchers to embed PPI in their work. We call on research institutions to invest in training, infrastructure and dedicated PPI staffing. And we call on the Parkinson's research community to share its experiences of PPI, particularly what has worked, what has not, and what has been learned, so that the evidence base grows and the field advances together.

## Data Availability

Data sharing is not applicable to this article as no datasets were generated or analysed during this study.
